# Long non-coding RNA Databases in Cardiovascular Research

**DOI:** 10.1016/j.gpb.2016.03.001

**Published:** 2016-04-02

**Authors:** Frank Rühle, Monika Stoll

**Affiliations:** 1 Institute of Human Genetics, Genetic Epidemiology, University of Muenster, 48149 Muenster, Germany; 2 Cardiovascular Research Institute Maastricht (CARIM), Genetic Epidemiology and Statistical Genetics, Maastricht Center for Systems Biology (MaCSBio), Maastricht University, 6211 LK Maastricht, The Netherlands

**Keywords:** Database, Non-coding, lncRNA, Gene regulation, Cardiovascular disease, *ANRIL*

## Abstract

With the rising interest in the regulatory functions of long **non-coding** RNAs (**lncRNAs**) in complex human diseases such as **cardiovascular diseases**, there is an increasing need in public **databases** offering comprehensive and integrative data for all aspects of these versatile molecules. Recently, a variety of public data repositories that specialized in lncRNAs have been developed, which make use of huge high-throughput data particularly from next-generation sequencing (NGS) approaches. Here, we provide an overview of current lncRNA databases covering basic and functional annotation, lncRNA expression and regulation, interactions with other biomolecules, and genomic variants influencing the structure and function of lncRNAs. The prominent lncRNA antisense noncoding RNA in the INK4 locus (***ANRIL***), which has been unequivocally associated with coronary artery disease through genome-wide association studies (GWAS), serves as an example to demonstrate the features of each individual database.

## Introduction

Although substantial genetic heritability is estimated for complex cardiovascular diseases, *e.g.*, 40% in coronary artery disease (CAD) [1], and extraordinary efforts have been made in genome-wide association studies (GWAS) and meta-analyses to identify genetic variants leading to CAD, only a small fraction of genetic variance of CAD of ∼10% can be explained by genetic variants in protein-coding genes [2]. Additionally, the high proportion of GWAS associations in non-coding genome regions contradicts the simple view of potentially-deleterious protein mutations and indicates a complex regulatory network driven by non-coding RNAs (ncRNAs) [3], [4]. Since only 1% of the mammalian genome is translated into proteins, but approximately 85% of the genome is transcribed into RNA, ncRNAs potentially represent an additional layer of epigenetic regulation. Especially long ncRNAs (lncRNAs, RNA >200 nucleotides in length) provide a wide range of regulatory functions including interactions with DNA, RNAs, and proteins [5], [6].

For instance, the lncRNA X-inactive-specific transcript (*XIST*) directly binds to the polycomb repressive complex 2 (PRC2) and thereby downregulates the entire chromosome during X-chromosome inactivation [7]. Other lncRNAs influence gene activity by RNA-directed chromatin remodeling [8], RNA-directed DNA methylation [9] or as activator or repressor molecules for transcription factors (TFs) [10], [11]. By recruiting splicing factors or by masking splice junctions of mRNAs, lncRNAs can influence alternative splicing of coding genes [12]. Various lncRNA interactions with microRNAs (miRNAs) impact mRNA stability by masking miRNA-responsive elements or by competing for miRNA binding in competing endogenous RNA (ceRNA) networks [13], [14]. Additionally, discrimination between coding and non-coding genes is sometimes ambiguous, because functional lncRNA transcripts containing open reading frames may also be translated to (small) proteins [15].

Dysregulated expression or function of lncRNAs has been recognized to contribute to heart development and complex cardiovascular diseases [6]. For instance, transcript levels of the antisense noncoding RNA in the INK4 locus (*ANRIL*, alias *CDKN2B-AS1*) lncRNA, which is encoded on chromosome 9p21 at the strongest genetic susceptibility locus for CAD, are directly correlated with the severity of atherosclerosis [16]. The locus at chromosome 5q31 carrying the non-coding steroid receptor RNA activator (*SRA1*) as well as human leukocyte antigen (HLA) complex group 22 (*HCG22*) at chromosome 6p21 have been significantly associated with dilated cardiomyopathy (DCM) [17], [18]. The myocardial infarction (MI)-associated transcript (*MIAT*) encoded on chromosome 22q12 is implicated to play a role in MI [19].

To discover potentially harmful lncRNA functions, it is important to understand the complex interaction networks of these molecules. In general, expression of lncRNAs is more specific for cell type and developmental stage than that of protein-coding genes [20]. Functional prediction of lncRNAs is more difficult than, *e.g.*, that for smaller miRNAs, because function of a lncRNA is not solely determined by its nucleotide sequence, but by the resulting secondary structure enabling it to interact with other biomolecules [21]. This is supported by the fact that lncRNA sequences are less conserved than miRNAs or protein-coding genes except for their promoter regions [22]. Genomic variants in lncRNA sequences may induce abnormal expression and function of their harboring lncRNAs, *e.g.*, by gaining or losing binding sites for interaction partners or by altering the secondary structure even at distant positions of the RNA molecule, possibly explaining part of genetic susceptibility to certain diseases [23]. Many of the aforementioned disease-associated lncRNAs like *ANRIL*, *MIAT*, and *HCG22* have gene variants, whose structural impact is not yet understood. Generally, there is a large gap between the number of identified lncRNAs and their known functional impact.

Therefore there is a need for comprehensive lncRNA databases to utilize the huge experimental datasets from current high-throughput technologies joined by massively parallel sequencing such as RNA-Seq, chromatin immunoprecipitation (ChIP)-Seq, RNA immunoprecipitation (RIP)-Seq, cross-linking immunoprecipitation (CLIP)-Seq or chromatin isolation by RNA purification (ChIRP)-Seq [24]. In addition to the main genomics data portals from NCBI, EMBL and UCSC, which also provide data on non-coding genes, several specialized databases have been developed that collect and integrate data in the context of lncRNAs [25], [26]. All databases discussed here are accessible via a web-based interface and have been published in peer-reviewed journals (Table 1 and Table 2). Apart from these, there are further data repositories with downloadable data files like the Human lincRNA Catalog from Broad Institute [20]. While some databases have been performing well for several years, many specialized databases have been developed in very recent time, highlighting the strong momentum of this research field.

**Figure 1:**
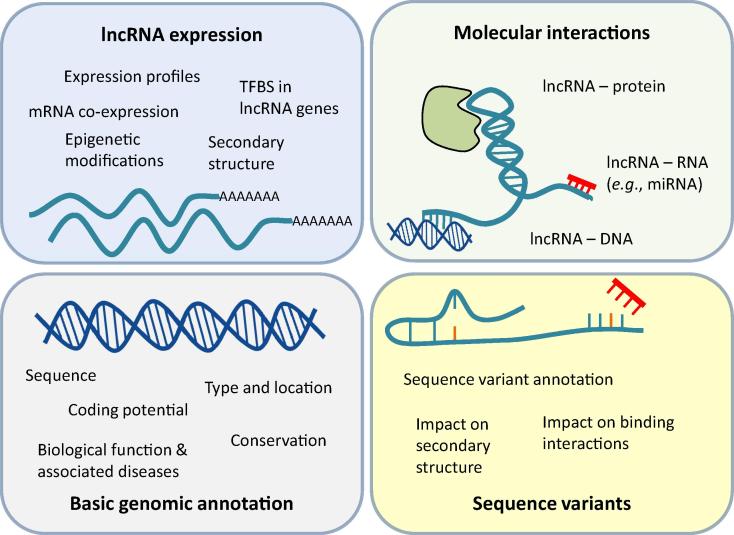
**Types of information curated in lncRNA databases** The available data have been grouped into four categories: basic genomic annotation, lncRNA expression, molecular interactions, and sequence variants. Databases for these kinds of information are listed in Table 1. lncRNA, long non-coding RNA; TFBS, transcription factor-binding site; miRNA, micoRNA.

In the following, we will give an overview of selected databases for different kinds of lncRNA-related information (Figure 1). The suggested analysis outline is exemplified by the CAD-related human lncRNA *ANRIL*. All specifications of database contents and query results refer to status of March, 2016 (Table 1 and Table 2). To start a query for a lncRNA of interest, basic information about lncRNA type, chromosomal location, nucleotide sequence, expression profiles, and functional annotation may be retrieved at NONCODE [27], [28], [29], [30], [31] and lncRNAdb [32]. Roughly, the classification of lncRNA types is based on their genomic context concerning sense, antisense, bidirectional, intergenic (lincRNAs), or intronic lncRNAs [33]. Known biological functions such as gene ontology (GO) annotation and disease associations are documented in LncRNADisease [34]. In addition to providing lncRNA expression profiles, lncRNAtor [35] offers co-expression data for protein-coding genes to identify potential functional connections between coding and non-noncoding transcripts. To gain insights in regulation of lncRNA expression, ChIPBase [36] contains information on TFs that regulate the expression of non-coding genes. In the next step, the interactions of lncRNAs with other biomolecules may be examined by using starBase [37] and NPInter [38]. These databases provide experimentally-validated data on interactions with proteins, DNA, and other RNA types, especially miRNAs. Finally, genomic variations within the lncRNA gene sequence can be analyzed to explore their potential functional influence on the lncRNA transcript using lncRNASNP [39].

## NONCODE 2016

NONCODE was first published in 2005 as an integrated knowledge database of ncRNAs [27] and has repeatedly been updated since then [28], [29], [30], [31]. Its latest version NONCODE 2016 offers data for 16 species including 167,150 human lncRNAs [31]. In addition to lncRNA class, chromosomal location, sequence, Coding-Non-Coding Index (CNCI) for protein coding prediction and expression profiles, the database included conservation annotation and disease association as new features in its latest version. The collected data are curated from published literature and include input from other public databases such as Ensembl [40], RefSeq [41], lncRNAdb [32], and GENCODE [42]. The database established a lncRNA nomenclature consisting of “NON”, a three character code that specifies the species, “T” or “G” for transcript or gene, respectively, followed by six sequential numbers and a version number where applicable. For *ANRIL* (NONHSAG051899), we find 22 transcript isoforms of type ‘antisense’ on chromosome 9, which are mostly expressed in lung, lymph nodes, prostate, skeletal muscle, and white blood cells. However, although this molecule has been linked in the literature to cardiovascular diseases and several other pathologies, we don’t find any disease association for *ANRIL* in NONCODE.

## lncRNAdb v2.0

Unlike NONCODE, lncRNAdb [32], [43] contains only functionally-annotated entries manually curated from referenced literature. lncRNAs that have been associated with diseases but have not been further characterized by knockdown or overexpression experiments, are not included in the repository. To date, the database contains 295 functionally-annotated lncRNAs covering 71 species, including 183 lncRNAs annotated in human. The database gives information on lncRNA type, sequence, chromosomal and subcellular localization, characteristics and functional annotation complemented by literature references, evolutionary conservation, interactions with other biomolecules, as well as expression profiles based on the Illumina body map [44]. In lncRNAdb, *ANRIL* is implicated in a range of complex diseases including cancer, T2D, and coronary heart disease. Its expression in tissues and cell types that are affected by atherosclerosis, such as peripheral blood mononuclear cells, whole blood, and atherosclerotic plaque tissue, is directly correlated with the severity of atherosclerosis. Functional interaction of *ANRIL* is described for chromobox 7 (CBX7), a component of the PRC1 [8]. According to the deposited expression profiles, *ANRIL* is mainly expressed in colon tissue.

## LncRNADisease

LncRNADisease [34] collects experimentally-validated disease associations of human lncRNAs extracted from the literature. By now, the database contains more than 1000 lncRNA–disease annotations including 321 lncRNAs and 221 diseases from about 500 publications. LncRNADisease also curates 475 entries of validated lncRNA interactions with other biomolecules including protein, RNA, and DNA. In addition, a computational method has been developed to predict new potential disease associations for a given lncRNA based on its genomic context. The database can be queried for either lncRNAs or diseases. Since *ANRIL* belongs to the well-annotated lncRNAs contributing to disease, we find a total of 134 lncRNA–disease associations described in 65 publications and affecting 37 disease phenotypes including CAD, MI, T2D, and several cancer types. Additionally, 25 interaction entries of *ANRIL* with 9 different biomolecules are annotated, including co-expression and regulatory interactions with its protein-coding counterparts *CDKN2A* and *CDKN2B* which encode cyclin-dependent kinase inhibitor 2A and B, and protein binding interaction with PRC1 and PRC2.

## lncRNAtor

Expression data from 243 RNA-Seq experiments comprising 5237 samples of various tissues and developmental stages have been collected from the public databases, including Gene Expression Omnibus (NCBI GEO) [45], ENCODE [46], modENCODE [47], and The Cancer Genome Atlas (TCGA) [48], and are updated on an annual basis [35]. The lncRNA compendium was taken from Ensembl [40], HUGO Gene Nomenclature Committee (HGNC) [49], Mouse Genome Database (MGD), [50] and lncRNAdb [32], and comprises a total of 21,575 lncRNA genes from human, mouse, zebrafish, fruit fly, worm, and yeast. In addition to visualizing tissue-specific expression profiles of lncRNAs, expression data can be searched for co-expression of mRNAs to identify putative lncRNA–mRNA pairs. Functional investigation of lncRNAs is complemented by CLIP-Seq and RIP-Seq data included from public data repositories to identify potential protein–lncRNA interactions. As most of the included human datasets are cancer-related, we find *ANRIL* to be upregulated in several cancer types compared to normal tissue, namely in kidney- and liver-related carcinoma. Co-expression analysis in a dataset of kidney renal clear cell carcinoma reveals *ARF5* (encoding ADP ribosylation factor 5) as highly-correlated coding gene, which possibly interacts with *ANRIL* in *trans*. Protein interactions are displayed for 12 proteins, including argonaute 2 (AGO2, RNA-induced silencing complex catalytic component) and DiGeorge syndrome critical region 8 (DGCR8), indicating potential involvement in miRNA regulation.

## ChIPBase

ChIPBase [36] aims at analysis of the transcriptional regulation of lncRNAs and miRNAs. It contains TF–lncRNA and TF–miRNA regulatory relationships identified by data coming from 543 ChIP-Seq experiments for 252 different TFs retrieved from the respective research articles and the NCBI GEO [45], ENCODE [46], and modENCODE [47] databases. The collection comprises diverse tissues and cell lines from human, mouse, dog, chicken, fruit fly, and nematodes (TF–lncRNA relationships are not available for dog, chicken, and nematode). Additionally the database is complemented by human expression profiles from 22 tissues. ChIPBase can be queried by lncRNAs, miRNAs or TFs, and the results are visualized by an integrative genome browser. For *ANRIL*, ChIPBase displays experimentally-supported binding sites of 18 different TFs in human, including androgen receptor (AR), v-ets avian erythroblastosis virus E26 oncogene homolog (ERG), and signal transducer and activator of transcription 1 (STAT1).

## NPInter v3.0

NPInter v3.0 [38], [51] provides experimentally-verified functional interactions between ncRNAs and other biomolecules such as proteins, RNAs and genomic DNA. Interaction data for 23 different species (mainly human and mouse) are collected from literature datasets and related databases such as lncRNADisease [34]. ncRNAs are screened against NONCODE [31], which serves as ncRNA reference database. The majority of included data stems from systematic identification of protein-binding sites by CLIP-Seq experiments, while other interactions such as ncRNA–RNA and TF–ncRNA are obtained mainly from interaction studies on individual ncRNAs. NPInter classifies all interactions as ‘binding’, ‘regulatory’, or ‘co-expression’. Every interaction entry includes a description of the kind of interaction and the interacting partner, complemented by the source of experimental data and the corresponding PubMed ID. Additionally, computational tools have been added to its latest version to predict further RNA–RNA and RNA–protein interactions. For *ANRIL* (query for NONCODE ID NONHSAG051899), NPInter displays 73 interactions, including RNA−DNA and RNA−protein binding interactions to its protein-coding counterparts *CDKN2A* and *CDKN2B*, regulatory interaction with miRNA hsa-miR-106a, and binding to the TF STAT1.

## starBase 2.0

starBase 2.0 [37], [52] collected 111 CLIP-Seq data sets from various tissues and cell lines generated by 40 independent studies from the NCBI GEO [45] to explore protein–RNA and various RNA−RNA interactions as well as ceRNA regulatory networks involving miRNAs, lncRNAs and mRNAs. miRNA data and gene annotations were retrieved from miRBase [53], GENCODE [54], Ensembl [40], and RefSeq [41], respectively. miRNA target sites on lncRNAs are predicted by miRanda [55] and subsequently filtered for CLIP-supported interactions. For *ANRIL* (query for *CDKN2B-AS1* because the gene symbol *ANRIL* is not found by starBase), 21 human miRNA–lncRNA interactions are annotated in the database. Interestingly, these do not include hsa-miR-106a identified by NPInter mentioned above. In addition, expression profiles are given for miRNAs and lncRNAs if available. When searching for ceRNA networks involving *ANRIL* and a minimum of 5 common miRNAs, we find *TMEM41A* coding for transmembrane protein 41A to be part of the network.

## lncRNASNP

lncRNASNP aims at the influence of genetic variants on the expression and function of the encoded lncRNAs. This influence may arise from gain or loss of binding sites for miRNAs or induction of conformational changes within the secondary structure of a lncRNA. Therefore, lncRNASNP collected SNP data and lncRNAs from dbSNP [56], LNCipedia [57], and NONCODE [31], respectively. Changes in secondary structure are predicted by RNAfold [58] based on the minimal free energy of the alternative transcript sequence. miRNA sequences were downloaded from mirBase [53] and are used to predict target sites on lncRNAs using the TargetScan [59] and miRanda [55] algorithms. Furthermore, experimentally-supported lncRNA–miRNA interactions from starBase [37] and disease associations from the National Human Genome Research Institute (NHGRI) GWAS Catalog [60] are embedded in the database. lncRNASNP is divided into human and mouse sub-databases and can be queried for SNPs, lncRNAs, miRNAs, or genomic regions. It returns 17 transcripts for *ANRIL* (query for *CDKN2B-AS1* because the gene symbol *ANRIL* is not found by lncRNASNP). Transcript *CDKN2B-AS1-001* contains 20 SNPs and 90 predicted miRNA-binding sites. Two binding sites are gained due to alternative SNP alleles while 9 binding sites are lost. The secondary structures of wild type and variant sequence can be visualized for each SNP of interest.

## Other resources

In addition to the data repositories presented above, several other public data resources for lncRNA research are listed in Table 1 and Table 2. For instance, LNCipedia [57], [61] summarizes 111,685 human lncRNA transcripts from Ensembl [40], lncRNAdb [32], NONCODE [31], RefSeq [41], the Human lincRNA Catalog [20], and two further datasets published by Hangauer et al and Nielsen et al [62], [63]. It offers transcript and structure information as well as computational scores for protein-coding potential and miRNA-binding sites. lncRNome [64] also provides a range of general annotations for sequence, structure, function, variation, and epigenetic modifications for more than 17,000 human lncRNAs derived from public databases. For protein–lncRNA interactions, the database included published photoactivatable-ribonucleoside-enhanced CLIP (PAR-CLIP) experiments and computational prediction methods. More specialized databases exist for evolutionary conservation (PhyloNONCODE [65]) and functional annotation based on ceRNA interaction networks (Linc2GO [66]). Expression profiles of non-coding and coding genes are further available from RNA-Seq experiments (lncRNAMap [67]) and microarray platforms (NRED [68]). Co-LncRNA identifies co-expressed coding genes from RNA-Seq data, which are then functionally annotated. Potential influence of lncRNAs on target gene expression may be identified with LncRNA2Target [69], which contains manually-curated differential expression data from 217 lncRNA knockdown or overexpression experiments for human and mouse. Further interaction data between lncRNAs and other biomolecules can be found in LncReg [70], DIANA-LncBase [71], or lncRNAMap [67]. Many of these databases provide information for human and murines only, but there are also databases specialized in other model organisms such as zflncRNApedia [72] for zebrafish (*Danio rerio*) or PLncDB [73] for *Arabidopsis thaliana*.

## Concluding remarks

The growing number of interconnected lncRNA databases reflects the immense research interest in lncRNAs, which is increasingly gaining momentum in the quest to understanding the (dys)function of biomolecular networks potentially contributing to complex human diseases [74]. Current high-throughput technologies joined with massive parallel sequencing generate data for non-coding transcripts at an unprecedented scale. To date, there is still a strong disconnection between the large number of identified transcripts and the small amount of lncRNA functional data, which is illustrated best by two of the most cited lncRNA databases, NONCODE and lncRNAdb. While NONCODE contains as much as 167,150 known human lncRNA transcripts, lncRNAdb is dedicated to functionally-characterized lncRNAs, restricting its content to 183 human lncRNAs. However, even for well-characterized lncRNAs, such as *ANRIL*, further investigation is warranted. Despite the wealth of information from public databases, the exact mechanisms of *ANRIL* functionality remain enigmatic. Another drawback are occasional discrepancies across databases for similar queries, which force researchers to use and compare several databases [25]. When choosing a database, researchers should also assure that the database of interest is curated and regularly updated as novel information becomes available. For instance, the Functional lncRNA Database [75] was not considered for this review since it has last been updated in March 2012. Nevertheless, current databases offer valuable resources for integration and interpretation of various kinds of experimental lncRNA data. This is essential for understanding the function and relevance of these versatile molecules and may pave the way to new translational applications in cardiovascular research.

## Competing interests

The authors declare that there are no conflicts of interests.

## Figures and Tables

**Table 1:** Overview of current lncRNA databases

*Note:* Included data types are indicated by colored dots. The color code represents a graduation of databases in three equally-sized groups based on the number of citations per year for the initial and update database publications (red: upper third with most cited databases; blue: middle third; green: lower third; citations were retrieved from Scopus database in March, 2016). Some of the ‘green’ databases were published very recently and therefore have not yet been cited. Data types are grouped in functional categories indicated by background colors. Gray: basic genomic annotation; blue: lncRNA expression; green: molecular interactions; yellow: sequence variants. Accessible web links to all databases are given in Table 2. *Hsa*, *Homo sapiens*; *Mmu*, *Mus musculus*; *Dre*, *Danio rerio*; *Ath*, *Arabidopsis thaliana*; *Cel*, *Caenorhabditis elegans*.

**Table 2:** Web links and data content of the presented lncRNA databases

**Database**	**Weblink**	**Data content**	**Refs.**
ChIPBase 1.1	http://deepbase.sysu.edu.cn/chipbase/	543 ChIP-Seq datasets	[36]
C-It-Loci	http://c-it-loci.uni-frankfurt.de/	119 RNA-Seq datasets	[76]
Co-LncRNA	http://www.bio-bigdata.com/Co-LncRNA/	241 RNA-Seq datasets	[77]
DIANA-LncBase	http://carolina.imis.athena-innovation.gr/index.php?r=lncbasev2	CLIP-validated and predicted miRNA targets on lncRNAs	[71]
GermlncRNA	http://germlncrna.cbiit.cuhk.edu.hk/	Germ cell-related expression data	[78]
Linc2go	http://www.bioinfo.tsinghua.edu.cn/~liuke/Linc2GO/index.html	Functional annotation of predicted RNA interactions	[66]
LincSNP	http://210.46.85.180:8080/LincSNP/home.jsp	5000 lincRNAs and 140,000 disease-associated SNPs	[79]
lnCeDB	http://gyanxet-beta.com/lncedb/	>25,000 lncRNA transcripts from GENCODE	[80]
LNCipedia 3.1	http://www.lncipedia.org/	111,685 lncRNA transcripts from literature and public databases	[57], [61]
LncReg	http://bioinformatics.ustc.edu.cn/lncreg/	1081 manually-curated lncRNA interactions	[70]
lncRNA2Function	http://mlg.hit.edu.cn/lncrna2function/	9625 lncRNAs and RNA-Seq data from 19 tissues	[81]
LncRNA2Target	http://mlg.hit.edu.cn/lncrna2target/	lncRNA–target association from knockdown/overexpression	[69]
lncRNAdb v2.0	http://lncrnadb.com/	295 lncRNA genes curated from literature	[32], [43]
LncRNADisease	http://cmbi.bjmu.edu.cn/lncrnadisease	>1000 literature-extracted lncRNA−disease annotations	[34]
lncRNAMap	http://lncrnamap.mbc.nctu.edu.tw/php/	RNA-Seq data from GEO and SRA	[67]
lncRNASNP	http://bioinfo.life.hust.edu.cn/lncRNASNP/	Predicted effects of SNPs in >30,000 lncRNA transcripts	[39]
lncRNAtor	http://lncrnator.ewha.ac.kr/index.htm	243 RNA-Seq studies from public databases	[35]
LncRNAWiki	http://lncrna.big.ac.cn/index.php/Main_Page	lncRNAs of GENCODE, NONCODE, LNCipedia, and lncRNAdb	[82]
lncRNome	http://genome.igib.res.in/lncRNome/	>17,000 lncRNAs from public databases	[64]
NONCODE 2016	http://www.noncode.org/	527,336 lncRNA transcripts from literature and public databases	[27], [28], [29], [30], [31]
NPInter 3.0	http://www.bioinfo.org/NPInter/	About 500,000 validated molecular interactions	[38], [51]
NRED	http://nred.matticklab.com/cgi-bin/ncrnadb.pl	Expression profiles from 8 microarray platforms	[68]
PhyloNONCODE	http://www.bioinfo.org/phyloNoncode/	Conservation annotation for >135,000 lncRNAs	[65]
PLncDB	http://chualab.rockefeller.edu/gbrowse2/homepage.html	>13,000 lncRNAs from tiling arrays and RNA-Seq	[73]
SNP@lincTFBS	http://210.46.85.180:8080/SNP_linc_tfbs/	5835 lincRNAs, 690 ChIP-Seq datasets, and 140,000 SNPs from dbSNP	[83]
starBase v2.0	http://starbase.sysu.edu.cn/	111 CLIP-Seq datasets	[37], [52]
TF2LncRNA	http://mlg.hit.edu.cn/tf2lncrna/analyze.jsp	22,531 lncRNA transcripts and 425 ChIP-Seq datasets	[84]
zflncRNApedia	http://genome.igib.res.in/zflncRNApedia/	ChIP-Seq and RNA-Seq data for 2267 zebrafish lncRNAs	[72]
